# Association between the Time of Length since Smoking Cessation and Insulin Resistance in Asymptomatic Korean Male Ex-Smokers

**DOI:** 10.1155/2017/6074760

**Published:** 2017-06-19

**Authors:** Ko-Woon Kim, Sung-Goo Kang, Sang-Wook Song, Na-Rae Kim, Jun-Seung Rho, Yun-Ah Lee

**Affiliations:** ^1^Department of Family Medicine, College of Medicine, The Catholic University of Korea St. Vincent's Hospital, Suwon, Republic of Korea; ^2^Health Promotion Center, College of Medicine, The Catholic University of Korea St. Vincent's Hospital, Suwon, Republic of Korea

## Abstract

**Aim:**

Smoking is a major risk factor for diabetes mellitus, mainly due to decreased insulin secretion and increased insulin resistance. However, there has been little research on the effects of smoking cessation period on changes in insulin resistance. In this study, we investigated the relationships between the length of time since smoking cessation period and insulin resistance in asymptomatic Korean male ex-smokers.

**Methods:**

A total of 851 male adults were included in this study. We considered several factors that can affect insulin resistance, and through multiple linear regression analysis, we assessed the effect the length of time since smoking cessation on insulin resistance in ex-smokers. Insulin resistance was represented as the insulin resistance index estimated by homeostasis model assessment.

**Results:**

HOMA-IR values showed a statistically significant negative correlation with the length of time since smoking cessation (*p* = 0.009) in ex-smokers. After performing multiple linear regression analysis using factors that could potentially influence insulin resistance, we found that waist circumference (*p* = 0.026) and the length of time since smoking cessation (*p* = 0.039) were independent predictors of HOMA-IR in asymptomatic male ex-smokers.

**Conclusion:**

The longer the smoking cessation period, the more the insulin resistance tended to decrease in asymptomatic Korean male ex-smokers.

## 1. Introduction

Smoking is a leading global cause of preventable death. Due to smoking, nearly 6 million people have died and hundreds of billions of dollars are spent annually worldwide to minimize smoking practices [1]. The negative effects of smoking lead to reduced quality of life and cause personal and national financial burdens.

As awareness of the importance of smoking cessation has emerged, countries around the world have implemented various smoking cessation policies. As a result of many nonsmoking policies, daily smokers, the percentage of the population aged 15 years and older declined from 19.1% in 2000 to 12.9% in 2014 in the United States of America, according to health data from the Organization from Economic Cooperation and Development (OECD) [2]. In Korea, daily smokers decreased from 20.0% in 2014 to 17.3% in 2015 according to the OECD health data and the Korea National Health and Nutrition Examination Survey (KNHANES) [2, 3]. This suggests that smoking cessation policies such as increased in cigarette prices and the expansion of nonsmoking areas in 2015 were effective at reducing the smoking rate. However, this level has not yet reached the level of developed countries. In 2012, the rate of smokers aged 15 and over in Korea was 6th among the 34 OECD countries (21.6%).

Smoking is a major risk factor for metabolic syndrome and cardiovascular disease [4–7]. Diabetes mellitus (DM) is one of the most common chronic diseases, with an increasing prevalence rate in the world [8], and the number of patients in Korea is approaching 4.8 million. It is likely that the number of individuals with insulin resistance is much greater than this estimate.

Smoking is also a major risk factor for diabetes because it decreases insulin secretion [9] and increases insulin resistance [10–12]. Many studies have shown that active smoking increases the prevalence and incidence of not only type 2 diabetes mellitus (DM) [13–15] but also glucose intolerance [16]. The causes of insulin resistance are not well defined, but both genetic and environmental factors including obesity, physical inactivity, and smoking may play a role. For example, obesity [17, 18] and physical inactivity [19, 20] are considered important risk factors for insulin resistance. Craig et al. found that smoking increases triglycerides (TG), decreases high-density lipoprotein cholesterol (HDL-C), and causes hyperinsulinemia and resistance of insulin-mediated glucose uptake [11, 21, 22].

Facchini et al. compared the prevalence of insulin resistance according to smoking status and showed that smoking was correlated with greater insulin resistance [11]. It has also been reported that smoking increases insulin resistance in a dose-dependent manner in patients with type 2 diabetes mellitus [23]. Moreover, there were almost no previous studies on changes in insulin resistance according to the length of smoking cessation period. Therefore, we investigated the relationship between the length of smoking cessation period and insulin resistance in asymptomatic Korean male ex-smokers in this study.

## 2. Methods

### 2.1. Subjects

**Figure 1:**
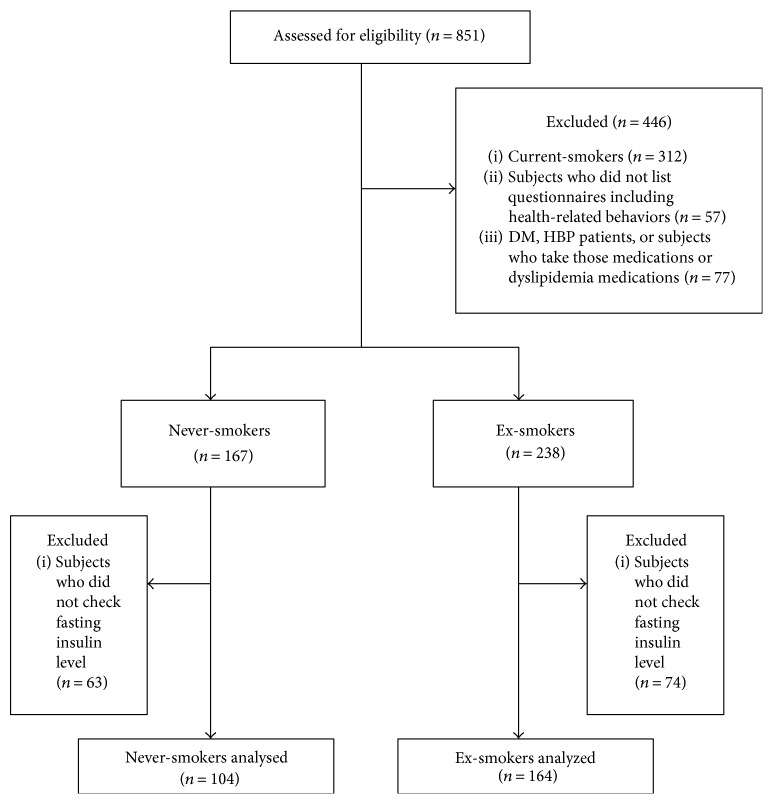
The flow chart of the study.

The initial study population included adult males who visited a university hospital in Gyeonggi province, Republic of Korea, for medical check-ups. At these check-ups, information regarding the cardio-ankle vascular index (CAVI) and ankle-brachial index (ABI) were routinely assessed. The subjects were screened before they were admitted to the study. Among the 851 questionnaires, 312 people were excluded for being current-smokers and 57 subjects were excluded for not providing health-related behaviors including smoking history. Also, 77 subjects were excluded who had been diagnosed with diabetes and hypertension or were receiving treatment for such diseases or dyslipidemia; 137 subjects were also excluded who had not been tested for insulin resistance (fasting insulin and/or fasting blood sugar). Thus, 104 never-smokers and 164 ex-smokers were included in this study (Figure 1).

### 2.2. Ethics Statement

This study was conducted in accordance with the ethical and safety guidelines of the Institutional Review Board (IRB) at The Catholic University of Korea St. Vincent's Hospital (IRB Approval number VC17RISI0019). Because we reviewed health screening data and medical record retroactively, this study was exempted from written informed consent requirements. The IRB approved this consent procedure.

### 2.3. Measures

Before undergoing a physical examination, subjects provided information about their smoking status, alcohol intake, and exercise habits using self-reported questionnaires.

Smoking status was classified as never-smokers and ex-smokers. Depending on what was listed in the questionnaire, alcohol consumption was defined as *No*, drinking <1 time per week or *Drinking*, ≥1 time per week; exercise was classified into *Regular*, ≥2 time per week and ≥30 minutes per time, *Irregular*, <2 time per week or <30 minutes per time, or *No*, no exercise. In this study, we measured factors that could affect insulin resistance including body mass index (BMI), waist circumference (WC), systolic blood pressure (SBP), diastolic blood pressure (DBP), TG, HDL-C, CAVI, and ABI.

Height and weight were measured with an automatic anthropometric instrument in an upright posture without shoes and socks, and BMI was calculated as body weight (kg)/height squared (m^2^). WC was measured at the midpoint between the lower rib and the upper iliac crest. Blood pressure was measured with an automatic manometer after a stable state for more than 5 minutes.

CAVI is not influenced by blood pressure but reflects the stiffness of all blood vessels, including the aorta, the femoral artery, and the tibial artery [24]. It was measured noninvasively using a Vasera VS-1000 system. CAVI can be calculated using the following formula:

(1) $$\begin{array}{c} CAVI=a\;\left[\left(\frac{2\rho }{\Delta P}\right)\times \ln\;\left(\frac{Ps}{Pd}\right)PWV^{2}\right]+b \end{array}$$

(Ps is SBP, Pd is DBP, Δ*P* = Ps − Pd, *ρ* is blood density, PWV is pulse wave velocity, and *a* and *b* are constants).

After measuring blood pressure in both arms and ankles, ABI was calculated according to the American Heart Association definition by dividing the higher systolic pressure of the right and left ankle by the higher systolic pressure of both arms.

Blood samples were collected on the day of examination after at least 8 hours of fasting for analyzing factors of metabolic syndrome including fasting plasma sugar (FBS), insulin, TG, and HDL-C levels for each sample by using an automatic clinical chemistry analyzer (Hitachi 7600; Japan, and Sysmex XE-2100; Japan).

Insulin resistance indicates that the response to insulin is less than normal under a given insulin concentration. In this study, we used the homeostasis model assessment of insulin resistance (HOMA-IR) and homeostasis model assessment of beta-cell function (HOMA-*β*). HOMA-IR and HOMA-*β* were calculated using the following formula:

(2) $$\begin{array}{c} HOMA‐IR=\frac{fasting\;glucose\;\left[mg/dL\right]\times insulin\;\left[\mu U/mL\right]}{405},\\ HOMA‐\beta =\frac{20\times fasting\;insulin\;\left[\mu U/mL\right]}{fasting\;glucose\;\left[mg/dL\right]/18-3.5}. \end{array}$$

### 2.4. Statistical Analysis

The data were analyzed using the Statistical Package for Social Sciences (SPSS), version 18.0 for Windows. Using the chi-squared test, the incontinuous variables in each group were compared. The continuous variables in each of the two groups were compared using the *t*-test. Because the standard deviation of HOMA-IR and HOMA-*β* was large compared to the mean and not normally distributed, we used nonparametric analysis such as Mann–Whitney or Kruskal-Wallis test. We performed multivariate regression analysis to evaluate factors that affected insulin resistance. *p* values less than 0.05 were considered statistically significant.

## 3. Results

### 3.1. Characteristics of Subjects

Clinical and metabolic characteristics of subjects are listed in Table 1 by smoking status. The total number of subjects was 268; 104 (38.8%) were never-smokers and 164 (61.2%) were ex-smokers. No statistically significantly differences were observed between never-smokers and ex-smokers for clinical or metabolic parameters. The average duration of smoking cessation was 10.09 ± 6.88 years, the shortest period was 0.5 years, and the longest was 32 years.

### 3.2. Associations between HOMA-IR and Other Cofactors in Ex-Smokers

Although not statistically significant, the HOMA-IR values tended to decrease as the period of smoking cessation was longer when analyzed by classifying the smoking cessation period every 5 years (Table 2).

As a result of analyzing associations between HOMA-IR and other cofactors in ex-smokers, HOMA-IR values showed a statistically significant positive correlation with BMI, WC, SBP, DBP, and TG. Moreover, HOMA-IR values showed a statistically significant negative correlation with the time elapsed since smoking cessation (*p* = 0.009) and HDL-C (*p* = 0.003). No statistically significant correlation was found between HOMA-IR values and age, CAVI, or ABI (Table 3).

### 3.3. Factors That Affect Insulin Resistance in Ex-Smokers

After performing multiple linear regression analyses, with factors that can potentially influence insulin resistance, we found that WC and the time elapsed since smoking cessation were independent predictors of HOMA-IR in asymptomatic Korean male ex-smokers (Table 4). However, the duration of the smoking cessation period and HOMA-*β* values were not significantly correlated.

## 4. Discussion

Many studies have shown that smoking is associated with insulin resistance [10–12]. However, studies on the relationship between smoking cessation period and insulin resistance are rare. Given these findings, we assessed the association between the length of time since smoking cessation and HOMA-IR values (as an indicator of insulin resistance) in asymptomatic Korean male ex-smokers.

We found that HOMA-IR values had a statistically significant negative correlation with the duration of the period since smoking cessation in ex-smokers. After performing multiple linear regression analysis, only WC and the duration of time since smoking cessation showed a statistically negative correlation with HOMA-IR values in ex-smokers. According to the Korea Diabetes Status Report in 2016, the prevalence of impaired fasting glucose (IFG) in Korea is 24.8%, which means that one in every four people has prediabetes [25]. And, it is well known that smoking is a risk factor of DM. From this perspective, the results of this study are meaningful that long periods of smoking cessation might contribute to lowering the prevalence of diabetes, which might also be another basis for implementing smoking cessation policies.

In addition to these findings that the length of time since smoking cessation in ex-smokers was negatively correlated with insulin resistance, this study confirmed that WC is the most reliable predictor of insulin resistance. The findings of the present study are in line with many other studies. In fact, many studies found that current smokers are more likely to have higher WC [26–28] values than nonsmokers. However, Berlin et al. found that high WC values were less frequent among current smokers [29]. Similarly, some studies have assessed the relationship between smoking and waist-hip ratio (WHR); Visser et al. found that current-smokers had the highest WHR and never-smokers the lowest [30]. Barrett-Connor and Khaw found that smokers had a higher WHR than never-smokers and observed a dose-response relationship between increasing WHR and increasing number of cigarettes smoked in both sexes [31]. Further research is needed on the mechanism by which cigarette smoking is correlated with insulin resistance.

In our study, the HOMA-IR value decreased by 0.02 annually after quitting smoking, and it also decreased by 0.048 when waist circumference decreased by 1 cm. In other words, we could estimate that the decrease of HOMA-IR value at 10-year smoking cessation was almost similar to the effect of 4 cm decrease in waist circumference. In this respect, the effect of the length of time since smoking cessation on insulin resistance might be important in that decreased waist circumference is the most reliable indicator of insulin resistance reduction.

Early vascular alterations, such as increased arterial stiffness and vascular endothelial cell injury, might be responsible for the development of the metabolic syndrome [32, 33]. Smoking may indeed accelerate those pathways. Actually, increased arterial stiffness was seen in smokers with diabetes mellitus [34], hypertension [35], and dyslipidemia [36, 37]. In our study, on the contrary, CAVI and ABI did not show a statistically significant negative correlation with HOMA-IR in ex-smokers. These findings are contrary to baseline characteristic data about CAVI and ABI of ex-smokers and never-smokers, which suggests that CAVI and ABI might not be sensitive enough to measure the recovery of vascular damage during smoking cessation.

This study has a few limitations. First, it may be difficult to generalize the results of this study because the study population was not large and, because all subjects voluntarily visited the health promotion center, there might have been a selection bias. Also, because the participants in this study were Korean, these results might not directly apply people of other races. In fact, there was a study about that high nicotine concentration was associated with compromised insulin secretion among non-Hispanic Whites and non-Hispanic Blacks, but not among Mexican Americans and other Hispanics [38].

Second, women were excluded from this study. In Korean culture, women's smoking tends to be concealed and the reliability of women's smoking statistics from surveys may be poor. In fact, in the KNHANES (2008), the self-reported female smoking rate was 5.9%, but the smoking rate was 2.36 times higher according to urine cotinine (13.9%) [39]. Third, we did not consider smoking amount and duration of smoking in ex-smokers before smoking cessation.

Despite these potential limitations, the present findings support the conclusion that the length of time since smoking cessation could be an independent predictor of insulin resistance in asymptomatic Korean male ex-smokers. This finding implies that smoking cessation can delay the onset of DM, which indicates the importance of smoking cessation in the primary prevention of DM.

## Figures and Tables

**Table 1:** Baseline characteristics of the study population according to smoking status.

	Never-smokers (*n* = 104)	Ex-smokers (*n* = 164)	*p* value
Age (years)	54.09 ± 8.07	54.34 ± 7.50	0.797
BMI (kg/m^2^)	24.30 ± 2.69	24.58 ± 2.81	0.420
WC (cm)	86.42 ± 6.39	86.96 ± 6.53	0.500
SBP (mmHg)	128.84 ± 14.02	129.93 ± 14.29	0.540
DBP (mmHg)	77.24 ± 10.05	78.84 ± 9.47	0.191
Alcohol use			0.181
No	39 (44.8%)	48 (55.2%)
Yes^a^	65 (35.9%)	116 (64.1%)
Exercise			0.383
Regular^b^	38 (35.5%)	69 (64.5%)
Irregular^c^	37 (37.8%)	61 (62.2%)
No^d^	29 (46.0%)	34 (54.0%)
HDL-C (mg/dL)	45.00 ± 10.87	45.79 ± 9.25	0.524
TG (mg/dL)	130.92 ± 80.16	145.11 ± 97.46	0.215
FBS (mg/dL)	95.36 ± 14.33	97.13 ± 13.91	0.314
INSULIN (*μ*U/mL)	3.73 ± 4.57	4.03 ± 3.76	0.547
HOMA-IR	0.89 ± 1.07	0.98 ± 0.96	0.333
HOMA-*β*	44.32 ± 55.27	46.02 ± 43.23	0.643
CAVI	7.61 ± 0.99	7.50 ± 0.84	0.324
ABI	1.15 ± 0.07	1.15 ± 0.07	0.650

*p* value was calculated by *t*-test or Mann–Whitney test for continuous variables or chi-square test for incontinuous variables. Values are presented as mean ± SD or n (%). ^a^Drinking ≥1 time per week. ^b^Exercise ≥1 time per week and ≥30 minutes per time. ^c^Exercise <1 time per week or <30 minutes per time. ^d^No exercises. BMI: body mass index; WC: waist circumference; SBP: systolic blood pressure; DBP: diastolic blood pressure; FBS: fasting blood sugar; TG: triglycerides; HDL-C: high-density lipoprotein cholesterol; HOMA-IR: homeostasis model assessment of insulin resistance; CAVI: cardio-ankle vascular index; ABI: ankle-brachial index.

**Table 2:** HOMA-IR values according to 5 years smoking cessation categories in ex-smokers.

Nonsmoking period (years)	*N*	Mean ± SD	*p* value
0–5	52	1.28 ± 1.28	0.118
6–10	55	0.93 ± 0.87	
11–15	29	0.80 ± 0.61	
≥16	28	0.98 ± 0.96	

*p* value was obtained by Kruskal-Wallis analysis.

**Table 3:** The correlation coefficient (*r*) between HOMA-IR and other cofactors in ex-smokers.

	*r*	*p* value
Age (years)	−.038	0.625
BMI (kg/m^2^)	.456	<0.001
WC (cm)	.484	<0.001
Nonsmoking period	−.203	0.009
SBP (mmHg)	.220	0.005
DBP (mmHg)	.280	<0.001
HDL-C (mg/dL)	−.233	0.003
TG (mg/dL)	.350	<0.001
CAVI	−.056	0.479
ABI	.057	0.470

*p* value was calculated by Pearson correlation. BMI: body mass index; WC: waist circumference; SBP: systolic blood pressure; DBP: diastolic blood pressure; HDL-C: high-density lipoprotein cholesterol; TG: triglycerides; CAVI: cardio-ankle vascular index; ABI: ankle-brachial index.

**Table 4:** Multiple linear regression between HOMA-IR and other cofactors in ex-smokers.

Variables	*β*	*p* value
Age (years)	−.004	0.687
BMI (kg/m^2^)	.037	0.451
WC (cm)	.048	0.026
Alcohol	.275	0.076
Exercise	.154	0.091
Nonsmoking period	−.020	0.039
SBP (mmHg)	.002	0.812
DBP (mmHg)	.007	0.560
HDL-C (mg/dL)	−.005	0.528
TG (mg/dL)	.001	0.397
CAVI	−.034	0.664
ABI	.517	0.562

BMI: body mass index; WC: waist circumference; SBP: systolic blood pressure; DBP: diastolic blood pressure; HDL-C: high-density lipoprotein cholesterol; TG: triglycerides; CAVI: cardio-ankle vascular index; ABI: ankle-brachial index.
